# *Aedes aegypti* insecticide resistance underlies the success (and failure) of *Wolbachia* population replacement

**DOI:** 10.1038/s41598-019-56766-4

**Published:** 2020-01-09

**Authors:** Gabriela A. Garcia, Ary A. Hoffmann, Rafael Maciel-de-Freitas, Daniel A. M. Villela

**Affiliations:** 1 Laboratório de Transmissores de Hematozoários, Instituto Oswaldo Cruz, Fundação Oswaldo Cruz (IOC/FIOCRUZ), Rio de Janeiro, Brazil; 2 University of Melbourne, Parkville, Australia; 3 Programa de Computação Científica, Fundação Oswaldo Cruz (PROCC/FIOCRUZ), Rio de Janeiro, Brazil

**Keywords:** Ecological modelling, Evolutionary ecology, Viral infection, Ecology

## Abstract

Mosquitoes that carry *Wolbachia* endosymbionts may help control the spread of arboviral diseases, such as dengue, Zika and chikungunya. *Wolbachia* frequencies systematically increase only when the frequency-dependent advantage due to cytoplasmic incompatibility exceeds frequency-independent costs, which may be intrinsic to the *Wolbachia* and/or can be associated with the genetic background into which *Wolbachia* are introduced. Costs depend on field conditions such as the environmental pesticide load. Introduced mosquitoes need adequate protection against insecticides to ensure survival after release. We model how insecticide resistance of transinfected mosquitoes determines the success of local *Wolbachia* introductions and link our theoretical results to field data. Two *Ae*. *aegypti* laboratory strains carrying *Wolbachia* were released in an isolated district of Rio de Janeiro, Brazil: *w*MelBr (susceptible to pyrethroids) and *w*MelRio (resistant to pyrethroids). Our models elucidate why releases of the susceptible strain failed to result in *Wolbachia* establishment, while releases of the resistant strain led to *Wolbachia* transforming the native *Ae*. *aegypti* population. The results highlight the importance of matching insecticide resistance levels in release stocks to those in the target natural populations during *Wolbachia* deployment.

## Introduction

The emergence and reemergence of arboviral diseases around the world is a significant concern for public health. High human mobility across countries, urban landscapes with poor sanitary conditions, and climate change all favor arthropod vector range expansion^1–3^. Among arboviruses with continental-wide distribution, dengue (DENV), chikungunya (CHIKV), Zika (ZIKV) and yellow fever (YFV) have caused recent outbreaks in multiple countries including Brazil^4,5^.

These four arboviruses are overwhelmingly transmitted by *Aedes* mosquitoes, with *Ae*. *aegypti* as the principal vector^6–8^. *Aedes aegypti* is closely associated with urban environments, such that females blood feed mainly on human hosts, lay eggs in domestic containers around human dwellings and rest inside houses^9–11^.

Since there are not effective vaccines or specific antiviral drugs available to low-income populations for DENV, CHIKV and ZIKV, control strategies target *Ae*. *aegypti* populations^2,12^. A relatively new strategy involves *Wolbachia*, intracellular maternally transmitted endosymbionts present in around 50% of insect species^13,14^. This bacterium, when transinfected into *Ae*. *aegypti* mosquitoes, reduces transmission of arboviruses such as DENV, CHIKV^15^ and ZIKV^16^. Thus, *Wolbachia* can be used for both population replacement and suppression. In replacement-oriented releases, an *Ae*. *aegypti* population highly competent for arbovirus is replaced by *Wolbachia*-carrying mosquitoes with significantly lower vector competence. Meanwhile, in suppression-oriented releases, the use of strains posing severe fitness costs could crash *Ae*. *aegypti* populations^17^, or combine incompatible and sterile insect techniques by releasing *Ae*. *albopictus* males^18^. Currently, *Wolbachia* has been deployed over 14 countries, including a variety of landscape, climate, demography and socioeconomic urban settings^19–22^.

Transinfected *Wolbachia* can be established in wild populations because they produce a frequency-dependent advantage for infected females by inducing cytoplasmic incompatibility (CI). The CI phenotype produces severe cell cycle defects in the male pronucleus, resulting in early embryonic lethality in crosses between *Wolbachia*-infected males and uninfected females^23,24^. *Wolbachia* frequencies tend to increase when the frequency-dependent CI advantage exceeds frequency-independent costs, which may be intrinsic to the *Wolbachia*, such as reduction in fecundity^25^, lower likelihood of surviving under starvation^26^, or associated with the genetic background into which *Wolbachia* are introduced, such as a genetic background susceptible to insecticides. Resistance to insecticides itself is likely to produce a fitness cost; overexpression of a resistance-conferring gene may result in a trade-off that involves resource reallocation at the expense of metabolic and developmental processes^27,28^ and mechanisms involving target-site modification may lead to a partial loss of function of a gene^29–31^.

Insecticidal based control is one of the most common approaches used to suppress *Ae*. *aegypti* populations in disease-endemic areas and can target both adult and larval stage of mosquito life cycle. Many studies have shown low insecticide efficiency due to development of resistance in wild *Ae*. *aegypti* populations^32–36^. Mutations in the voltage sodium channel gene produce a phenotype known as knockdown resistance (*kdr*). These mutations give rise to pyrethroids (PY) resistance, which has been related to fitness cost in many insects including *Ae*. *aegypti*^28,30,37,38^. Considering *kdr* mutations are globally spread in *Ae*. *aegypti* populations^35^, the genetics of released individuals must match those of native mosquitoes to foster invasion^22,39^. Insecticide resistance might be particularly useful for introducing *Wolbachia* infections with substantial fitness costs. Hoffmann and Turelli^40^ proposed an approach to facilitate *Wolbachia* invasion through insecticide-resistance selection, where insecticide-resistant mosquitoes infected with *Wolbachia* are deployed into an area in which insecticide usage suppresses wild population and thus enhances invasion. However, this strategy would require a susceptible native population, which may be rare around the globe^35,41^.

Direct evidence of the importance of matching the genetic background of native mosquitoes was provided when releasing *Wolbachia*-carrying mosquitoes in an isolated population in Rio de Janeiro, Brazil, with insecticide-resistant populations^22^. Releases failed to lead to stable establishment *Wolbachia*-transinfected when the released transinfected strain was susceptible to pyrethroids, whereas it was successful in a subsequent release with resistant wMel-infected *Ae*. *aegypti*^22^. Here, we perform an analysis of likely success/failure given insecticide-resistance in the field and varying intensities of insecticide use in the local human population. We model different scenarios of insecticide use and resistance. First, we evaluate the fitness cost of a colony of *Ae*. *aegypti* infected with the *w*Mel *Wolbachia* strain maintained in laboratory for 18 generations (*w*MelBr), without insecticide pressure. Second, we study several different features on the likelihood of successful *Wolbachia* invasion. These are: (1) releasing *Wolbachia* in a mosquito with susceptible and resistant strains (the *w*MelBr and *w*MelRio strains from Garcia *et al*.^22^); (2) varying the insecticide use by local householders during the releases; (3) changing levels of insecticide resistance in *Ae*. *aegypti* wild populations; and (4) altering the fitness cost of *Wolbachia* and insecticide resistance. We identify scenarios in which insecticide resistance of wild *Ae*. *aegypti* populations challenge successful *Wolbachia* invasion.

## Results

### Quantifying the fitness cost due to insecticide resistance

**Figure 1:**
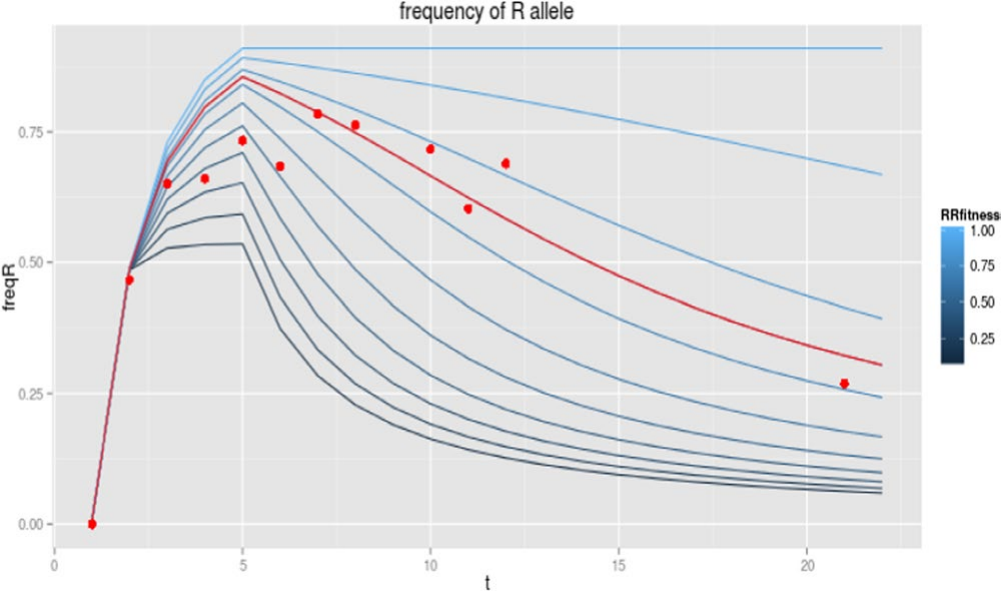
Observed and expected changes in the frequency of the resistance allele over time (laboratory generations). We assumed different fitness costs due to insecticide resistance based on the frequency of the *kdr* mutation, 1016Ile, along 18 generations when maintained under laboratory conditions, i.e., without insecticide pressure. Dots show the observed values and various curves constructed using the model show the expected frequencies when varying fitness of homozygous mosquitoes (factor *i*) from 0.1 to 1.0. The best fit using the lowest sum of residuals (curve in red color) has relative fitness *i* = 0.75.

We analyzed the frequencies of 1016Ile *kdr* mutations in the *w*MelBr colony without insecticide pressure across 18 generations. The frequency of the resistance gene decreased (Fig. 1), dropping from 0.75 to 0 after 18 generations. We estimate resistance fitness to be 0.75 (a fitness cost of 0.25). This value of parameter *i* was applied to the scenarios analyzed for *Wolbachia* invasion.

### Simulation scenarios

We considered *Wolbachia* releases and local wild *Ae*. *aegypti* mosquitoes resistant to the insecticides generally under two different sets of scenarios: deployment of *Wolbachia* infecting mosquitoes susceptible (*w*MelBr) or resistant (*w*MelRio) to insecticides. The intensity of insecticide application by local householders was also allowed to vary in these scenarios.

#### Scenario 1: Deployment of *Wolbachia* infecting a susceptible release strain (*w*MelBr) with wild resistant mosquitoes and insecticide pressures ranging from 0.0 to 0.9

**Figure 2:**
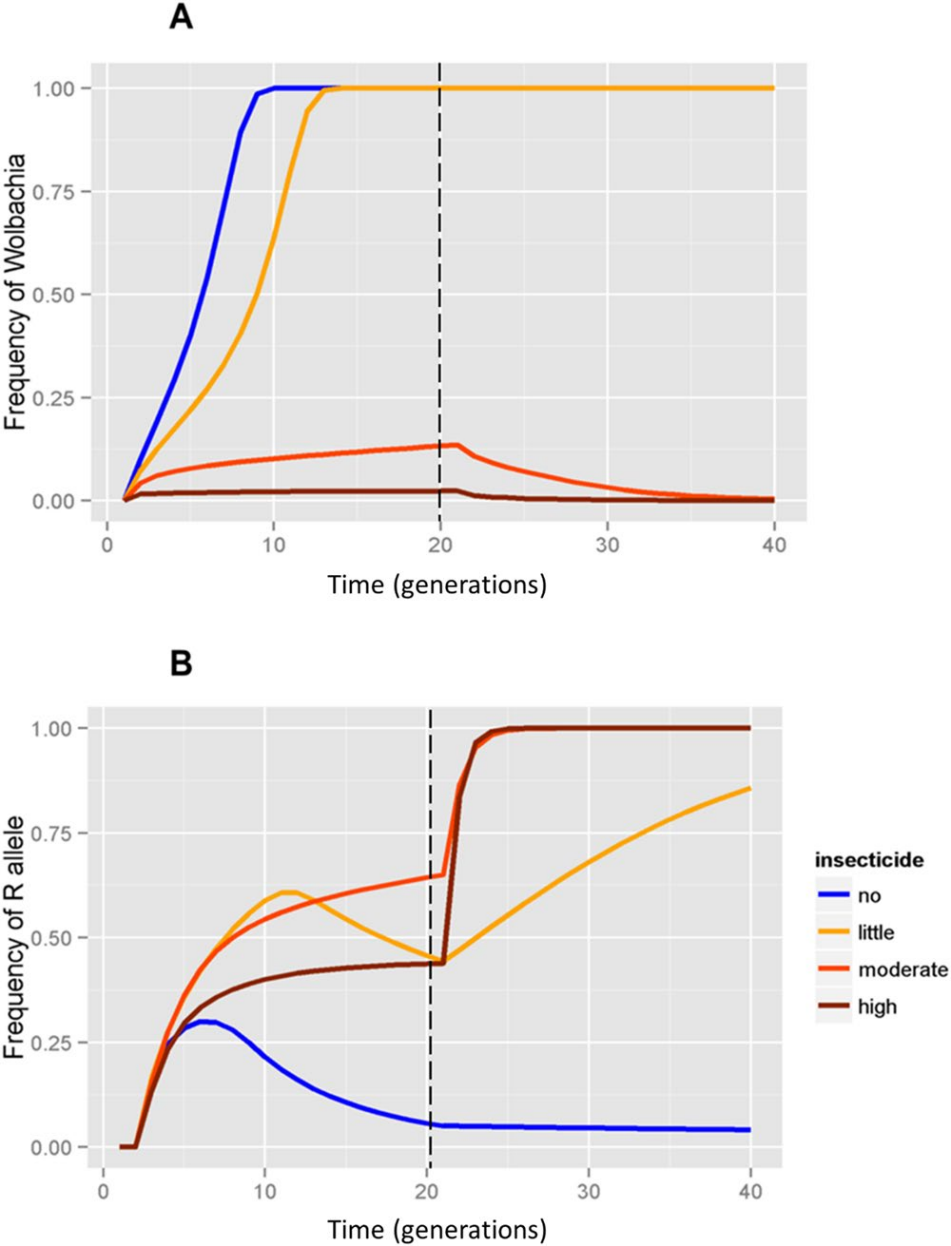
Releases of *Wolbachia* mosquitoes susceptible to insecticides. Frequency of (**A**) *Wolbachia* and (**B**) resistance alleles under different levels of insecticide use by local householders. Dashed line represents the end of releases.

Two outcomes were observed by releasing susceptible mosquitoes depending on whether there was no insecticide use (*s* = 0.0) or a low application intensity of *s* = 0.4 (Fig. 2A, blue and yellow line). As expected, in the absence of insecticide, *Wolbachia* invades rapidly. Additionally, the frequency of the R allele in the mosquitoes with *Wolbachia* increases due to introgression of the R allele in the first few generations. However, the frequency of R then decreases rapidly and is lost due to the continuous introduction of susceptible alleles through *Wolbachia* releases and due to fitness costs, resulting in a possible reversion of insecticide resistance status in the field after *Wolbachia* invasion (Fig. 2B, blue line). However, even an occasional insecticide application in the field selects R alleles in *Wolbachia* mosquitoes (Fig. 2B, yellow line).

*Wolbachia* does not invade when insecticide susceptible mosquitoes are released and local householders undertake moderate or high insecticide applications (Fig. 2A, red and brown line). In these two scenarios, *Wolbachia* frequency did not increase above 25%. The R alleles are rapidly selected in *Wolbachia* mosquitoes, despite the release of *Ae*. *aegypti* on a timely basis (Fig. 2B, red and brown).

#### Scenario 2: Deployment of mosquitoes carrying *Wolbachia on* resistant strain (*w*MelRio) with wild mosquitoes resistant and insecticide pressures ranging from 0.0 to 0.9

**Figure 3:**
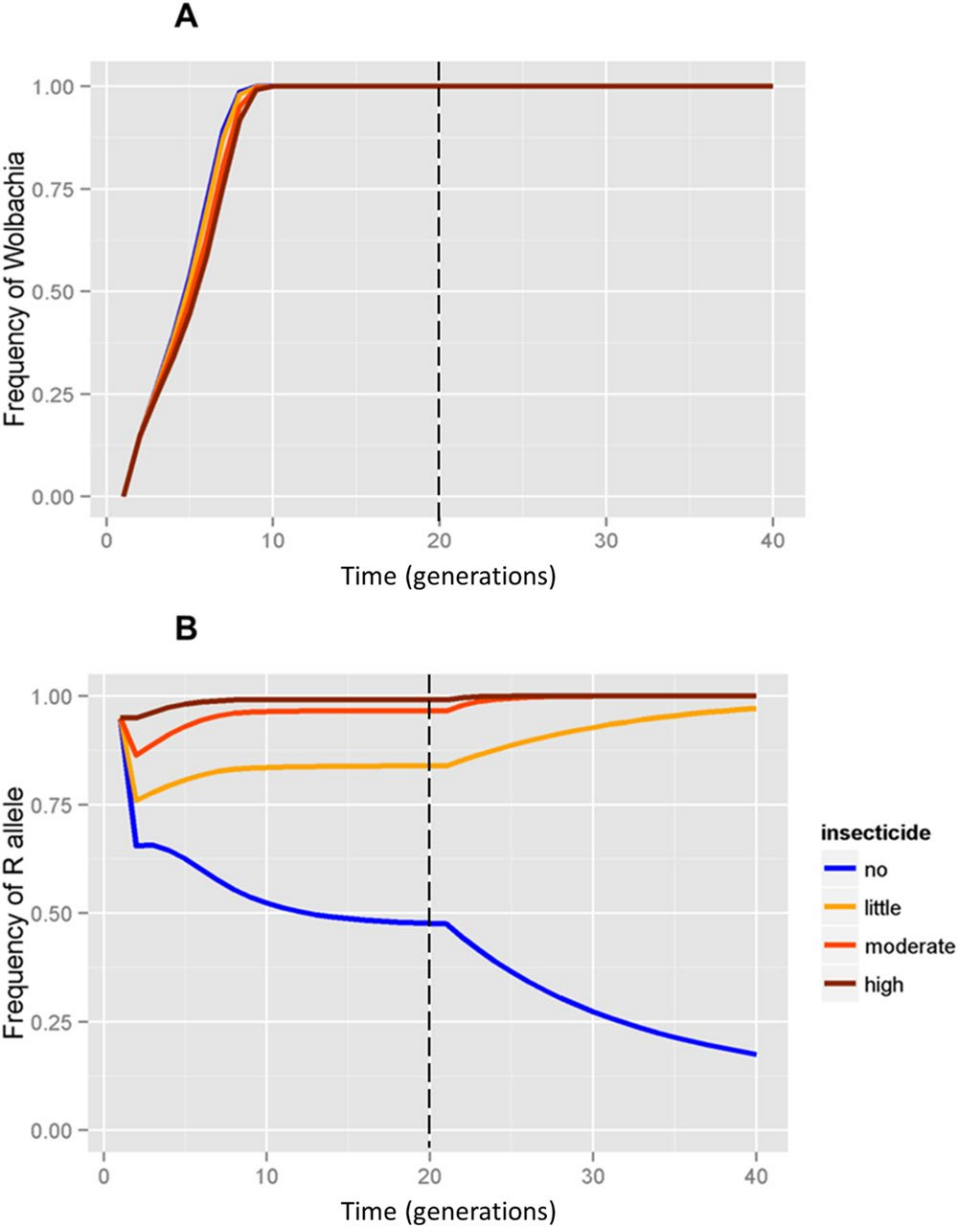
Releases of *Wolbachia* mosquitoes with comparable levels of insecticide resistance as those in the wild population. Frequency of (**A**) *Wolbachia* and (**B**) resistance alleles in field considering different levels of insecticide use by local householders. Dashed line represents the end of releases.

When releasing mosquitoes carrying *Wolbachia* on a strain resistant to insecticides, invasion always succeeds (Fig. 3A), regardless of variation in insecticide application intensity from s = 0.0 to s = 0.9 (Fig. 3B, blue, yellow, red and brown line). Insecticide applications did not alter the *Wolbachia* invasion profile, except for a minor tendency for faster *Wolbachia* invasion when insecticide intensity is low. In the absence of the insecticide, the frequency of the R allele decreases in the field (Fig. 3B, line blue), as shown in scenario 1, but due to the fitness cost of resistance in the absence of the insecticide, rather than the introduction of susceptible alleles by *Wolbachia* mosquitoes as in scenario 1. However, with any level of insecticide applications, the R allele reaches fixation in *Wolbachia* mosquitoes in the field (Fig. 3B, yellow, red and brown line). These results are in agreement with the proposal by Turelli and Hoffman^40^ showing invasion of resistant mosquitoes in places with susceptible wild mosquitoes.

## Discussion

We investigated how *Wolbachia* invasion success is influenced by the presence of insecticide resistance alleles in both the released and wild *Aedes aegypti* populations. Our model is based on the one by Hoffmann and Turelli^40^, but we take into account the fitness cost of pyrethroid resistance in order to analyze different scenarios of insecticide use and resistance. Considering *Wolbachia* deployment is undergoing expansion and the pyrethroid resistance in native *Ae*. *aegypti* populations is a worldwide phenomenon, *Wolbachia* success might be curtailed if the genetic background of released and local populations do not match, especially regarding insecticide resistance^22^. Therefore, results presented herein might inform release guidelines.

We estimated the fitness cost due to insecticide resistance at 0.25 over a generation time, based on empirical observations of *w*MelBr pyrethroid resistance allele loss over 18 generations. This finding points to an expected performance decrease in an insecticide-free environment. Since several vector control programs historically rely on chemicals, this would likely lead to distinct levels of resistance between wild and released populations^22^. Therefore, simulations of *Wolbachia* invasion must consider the insecticide resistance status of both released and natural populations.

Our results indicate that invasion of a susceptible strain is only possible if local householders use insecticides at low levels. Therefore, we set the conditions to determine how the frequency of pyrethroids application by local householders can affect *Wolbachia* invasion in the field. If no insecticide is used, *Wolbachia* invades faster and insecticide susceptibility status in field mosquitoes may increase rapidly, mainly due to the introduction of S alleles by *Wolbachia* mosquitoes. With the chosen model, we observe that although *Wolbachia* invades, R alleles are still selected even if local householders engage in a low level of insecticide applications. For moderate or high frequencies of insecticide application, susceptible *Wolbachia* released mosquitoes would die quickly as wild-resistant mosquitoes are at an advantage to survive and reproduce. In these situations, *Wolbachia* frequency would not increase above 25% in the field, and its frequency would remain low as mass releases stop. This scenario provides a likely explanation for the unsuccessful invasion of *w*Mel in Rio de Janeiro, since a susceptible strain (*w*MelBr) was released into a highly resistant field population. High insecticide pressure was likely based on information from local householders^22^. Field data showed that the *w*MelBr frequency reached 65% in the last week of release but sharply decreased afterwards when releases stopped. This partly fits scenario 1 (deployment of *w*MelBr susceptible mosquitoes into a native highly resistant population). In the field releases, between 12.5–24.2 mosquitoes were released per house weekly, for 20 consecutive weeks, and BG-Sentinel Traps were checked once a week, six days after release, and screened for *Wolbachia*^22^. Our model in scenario 1 suggests *w*MelBr frequency would not exceed 25% under moderate and high insecticide applications, but the higher frequency observed likely reflects weekly mass release of *w*MelBr-infected *Ae*. *aegypti*. The “real invasion” frequency expected from our model was probably reflected by the frequency of *w*MelBr a couple of weeks after releases stopped, which was around 20%. Garcia *et al*.^22^ hypothesized that given the high use of insecticides by households, only a small fraction of *w*MelBr-infected mosquitoes survived and reproduced, insufficient to overcome the threshold to promote invasion^42^, consistent with the modelled expectations presented here. Field releases were done on weekly basis, whereas our analytical results use generation time units, but these findings generally hold on different time scales.

The work by Hancock *et al*.^43^ evaluated how larval competition can modulate the invasion of *Wolbachia* at slower pace than often assumed. Also, a mathematical model structured by life stages analyzed the invasion of *Wolbachia*, also assuming diallelic locus model for insecticide resistance, among other traits evaluated^44^. Since overlapped generations are not observed, Hancock *et al*.^44^ rely on statistical models to obtain estimates. Such models exhibit the tradeoff between using overlapping and non-overlapped generations. Hancock *et al*., however, reported a number of 55 generations over a 4–5 month period. Since our model relies on generation time units, an initial intuition would require a long period of time if a generation takes multiple weeks. By contrast the overlapping generations may also signal invasion on time scales much shorter. Therefore, we believe that for this present study the most appropriate presentation is having a generation time unit, enabling elastic time scales, if necessary.

By contrast, when releasing a *Wolbachia* strain as resistant as the wild population (*w*MelRio), our results indicate that *Wolbachia* is able to invade irrespective of the intensity of insecticide application, and there is a decrease in the frequency of R alleles in the absence of insecticide which occurs more slowly than when a susceptible release strain introduces S alleles in field populations. The slow decrease in the frequency of R is consistent with studies that demonstrate slow insecticide resistance reversal when R alleles are at a high frequency^45,46^. For the other three intensities of insecticide application by local householders (0.4, 0.7, 0.9), selection maintains high frequencies of R alleles in the field. This matches what happened with a second round of releases in the same site in Rio de Janeiro which resulted in successful invasion by the *w*MelRio strain which had the same levels of insecticide resistance as the wild *Ae*. *aegypti* population^22^. It is worth noting that these results suggest that releasing resistant mosquitoes in places where susceptible ones dominate will also be successful even with little use of insecticide. This in fact is the proposal in the work by Turelli and Hoffman^40^ whose model demonstrated the success of *Wolbachia* invasion in this scenario. *Ae*. *aegypti* populations in various cities in Brazil already exhibit high levels of insecticide resistance^28^, but a strategy of releasing resistant *Wolbachia* mosquitoes in some areas that still have some susceptibility seems difficult to be adopted by authorities due to risk of raising resistance, if invasion does not happen successfully.

Insecticide resistance is frequently associated with a fitness cost on life-history traits such as larval development time and adult fecundity, longevity and locomotor activity^28,31,47–49^. The fitness cost due to insecticide resistance in our model was assessed by the rate of decrease of the *kdr* mutation in the strain *w*MelBr. This strain was backcrossed with Rio de Janeiro local populations^50^ and had a frequency of almost 70% of resistant genotypes. However, after eighteen generations with limited outcrossing (10% wild males every five generations) and no insecticide pressure, resistant genotypes dropped to 4%, resulting in a fitness loss estimate of 0.25. Brito *et al*.^30^ also observed 1016Ile *kdr* frequency decreasing to less than 30%, after 15 generations of *Ae*. *aegypti* without *Wolbachia* in laboratory cages, when starting from frequencies of 70% and 50% of *kdr* allelic frequency, consistent with the notion of a substantial fitness cost.

We assume in the model that insecticide resistance is governed by a single diallelic locus, with alleles denoted R and S^35,40^. There are, however, various factors which impact insecticide resistance, for instance metabolic resistance. Further study on modeling these factors are important to advance knowledge on the insecticide resistance, but certainly will be helpful to better understand *Wolbachia* invasion possibilities. We also considered that *w*Mel in *Ae*. *aegypti* has a small fitness cost, with minor alterations in larval competitive ability^26,51^, fecundity^25^ and fertility^22^. With these fitness costs and insecticide susceptibility in the release strain, invasion remains unlikely unless there is a sharp reduction in insecticide usage by local householders, which requires a significant effort from social scientists to change community behavior and vector control good practices. Successful releases will therefore likely require regular backcrossing of the release strain to maintain resistance in release material.

## Methods

### General model

The model is based on previous studies that have shown a fitness cost associated with PY target-site resistance, with a focus on two-allele representation of knockdown resistance based on 1016Ile *kdr* mutation^30,47,48,52,53^. Individuals can be classified by their resistance genotypes and *Wolbachia* infection state. Genotypes in a two-allele representation are given by RR, RS or SS for homozygous resistant, heterozygous and homozygous susceptible genotypes, respectively, as in Hancock *et al*.^44^ Insecticide susceptibility is typically a recessive trait^48^. The *Wolbachia* infection state is either uninfected (U) or infected (I). Without insecticides in the environment, homozygous-resistant mosquitoes have relative fitness given by a factor 1 – *i* compared to susceptible mosquitoes, hence a fitness cost given by *i*.

Turelli and Hoffmann^40^ developed a model in which a *Wolbachia* fitness cost *F*_*c*_ would apply over successive generations. We introduce in the present model a parameter to describe the fitness cost due to insecticide resistance. The model is designed from components that predict frequencies of resistance genotypes in successive generations and that consider varying intensities of insecticides application.

The first component evaluates frequencies *f(XX*,*WS)*_*t*_ of *XX* newly entering individuals (zygotes) at generation *t* where* XX* = {*RR*, *RS*, *SS*} and *WS* is the *Wolbachia* infection state, *WS* = {U, I}. The frequency of *Wolbachia* over generation *t* is described by *p*_t_ and the frequencies of R alleles in either *Wolbachia* mosquitoes or non-*Wolbachia* mosquitoes is given by *r*_*I*,*t*_ and *r*_*U*,*t*_, respectively.

These frequencies can be modeled by recursive equations such as

$$f{(RR,U)}_{t+1}=\frac{i{(1-{p}_{t})}^{2}{r}_{U,t}^{2}}{\bar{w}}$$

$$f{(RS,U)}_{t+1}={(1-{p}_{t})}^{2}2\,{r}_{U,t}\frac{1-{r}_{U,t}}{\bar{w}}$$

$$f{(SS,U)}_{t+1}=\frac{{(1-{p}_{t})}^{2}{(1-{r}_{U,t})}^{2}}{\bar{w}}$$

$$f{(RR,I)}_{t+1}=i\,{F}_{c}\,{p}_{t}\,{r}_{I,t}\frac{{p}_{t}\,{r}_{I,t}+(1-{p}_{t}){r}_{U,t}}{\bar{w}}$$

$$f{(RS,I)}_{t+1}={F}_{c}\,{p}_{t}\frac{2{p}_{t}\,{r}_{I,t}(1-{r}_{I,t})+(1-{p}_{t})({r}_{I,t}(1-{r}_{U,t})+{r}_{U,t}(1-{r}_{I,t}))}{\bar{w}}$$

$$f{(SS,I)}_{t+1}={F}_{c}\,{p}_{t}\frac{(1-{r}_{I,t})({p}_{t}(1-{r}_{I,t})+(1-{p}_{t})(1-{r}_{U,t}))}{\bar{w}}$$

where $\bar{w}$ is given by:

$$\begin{array}{c}\bar{w}=i{(1-{p}_{t})}^{2}{r}_{U,t}^{2}+{(1-{p}_{t})}^{2}2\,{r}_{U,t}(1-{r}_{U,t})+{(1-{p}_{t})}^{2}{(1-{r}_{U,t})}^{2}\\ \,+\,i\,{F}_{c}\,{p}_{t}\,{r}_{I,t}({p}_{t}\,{r}_{I,t}+(1-{p}_{t}){r}_{U,t})+{F}_{c}\,{p}_{t}({p}_{t}\,2\,{r}_{I,t}(1-{r}_{I,t})+(1-{p}_{t})({r}_{I,t}(1-{r}_{U,t})\\ \,+\,{r}_{U,t}(1-{r}_{I,t})))+{F}_{c}\,{p}_{t}\,(1-{r}_{I,t})({p}_{t}(1-{r}_{I,t})+(1-{p}_{t})(1-{r}_{U,t}))\end{array}$$

The frequencies of *Wolbachia* and the R allele in adults will be impacted by the use of insecticides. We assume that a fraction 1-*s* survives to mate and generate offspring. Therefore, insecticide intensity is defined in indirect manner, such that its impact is measured by the fraction of adult mosquitoes surviving as a decreasing function. The most intense insecticide intensity usage will impact in less numbers of adult mosquitoes surviving to generate offspring. This follows Equations 2.4 given by Turelli and Hoffmann^40^. In the field *s* reflects intensity of insecticide use, whereas in the laboratory for rearing *Wolbachia* individuals no insecticide is used, hence *s* = 0. The model with s = 0 is used to estimate a best fit for parameter *i*, based on frequency changes of the R allele when laboratory *Wolbachia* mosquitoes are maintained as closed populations or crossed with field males.

### Quantifying the fitness cost due to insecticide resistance (in laboratory conditions and without insecticide pressure)

Fitness costs due to *Wolbachia* presence and to insecticide resistance can be measured in the laboratory, where no insecticides are used during rearing of *Wolbachia* mosquito colonies. Estimates can be obtained from the model using a fixed *Wolbachia* fitness cost and varying costs due to insecticide resistance. We use the general model, with a particular approach that in the backcrossings we apply a frequency of resistance alleles equal to the one measured from field mosquitoes. Therefore, we expect an increase of frequency of resistance alleles during backcrossing generations. We vary the insecticide resistance cost *F*_*c*_ from 0.1 to 1 by increments of 0.01 and obtain for each cost value the sum of squared residuals considering the values predicted by the model and the frequencies observed in some of our lab generations (F5, F6, F7, F8, F9 and F18). The fitness cost due to the insecticide resistance is estimated as the cost producing the lowest sum of squared residuals.

### Parameters used in the *Wolbachia* invasion model

We analyze different scenarios varying in the initial levels of insecticide resistance among wild mosquitoes, as well as in the insecticide application during releases. In order to define scenarios, we also need initial conditions for the presence of *Wolbachia* in the field and for levels of insecticide resistance in the release population. For all simulations we consider *Wolbachia* to be absent in the field prior to releases. We consider a frequency *rU*_0_ of the R allele in the local population prior to releasing *Wolbachia* mosquitoes. This parameter represents the level of insecticides resistance gene in *Ae*. *aegypti* wild population that receive *Wolbachia* releases. Based on published data, we use a value of 0.95 in our analyses reflecting the fact that most wild mosquitoes are homozygous for resistance (RR)^22,48,52^.

Our model considers that *Wolbachia* mosquitoes are released on a periodic units of time for *n*_*rel*_ consecutive releases. In our analyses we considered *n*_*rel*_ = 20 releases in all simulations based on *Wolbachia* releases carried out in Rio de Janeiro from Sept/2014 to Jan/2015^22^. Each release of *Wolbachia* mosquitoes requires a release rate given by a ratio *r*_*rel*_ representing the number of released individuals divided by the total number of mosquitoes present *(released* + *local)* per unit of time. This parameter covers the density of wild mosquitoes and the number of *Wolbachia* mosquitoes released per unit of time. The unit of time used here is the time for a mosquito generation since the model is based on non-overlapping generations. We use an *r*_*rel*_ value of 0.10 based on releases in Brazil^22^ and for convenience we consider a timeframe of 40 mosquito generations (Table 1). Furthermore, our analysis indicates the frequency of the resistance allele within the total field population of *Wolbachia* mosquitoes, including the released mosquitoes (with releases lasting 20 units of time), plus field offspring, over the 40 generations period. Wild mosquitoes (without *Wolbachia*) were not taken into account due to a lack of initial gene flow from *Wolbachia* mosquitoes to the wild population, as a consequence of cytoplasmic incompatibility and complete maternal transmission^40^.

**Table 1:** Fixed parameters in the model with respective descriptions and values used in simulations.

Parameters	Description	Values	References
*i*	Fitness of homozygous resistant mosquitoes (0.0–1.0)	0.75	Brito *et al*. 2013^30^
*h*	Fitness factor for heterozygous mosquitoes (resistance nearly recessive)	0.8	Brito *et al*. 2018^48^
*F*_*c*_	Fitness of *Wolbachia*-carrying mosquitoes	0.8	Turley *et al*. 2013^25^; Hoffmann *et. al* 2014^54^; Ross *et al*. 2016^26^; Garcia *et al*. 2019^22^
*rU*_0_	Local population frequency of R (95%)	0.95	Linss *et al*. 2014^52^; Bellinato *et al*. 2016^55^; Brito *et al*. 2018^48^; Garcia *et al*. 2019^22^
*n*_*rel*_	Releases	20	Garcia *et al*. 2016^56^
*r*_*rel*_	Ratio of released individuals by the total number (released + local) per unit of time	0.10	Garcia *et al*. 2016^56^
*T*_*tot*_	Total number of generations	40	—

### Construction of potential invasion scenarios

Our scenarios consider the intensity of insecticide used by the local human population and the resistance of *Wolbachia* mosquitoes (Table 2). We first consider that insecticide intensity *s* varies in the simulation scenario. We consider some scenarios with no application (*s* = 0.0), low use (*s* = 0.4), moderate use (*s* = 0.7), or high insecticide use (*s* = 0.9). We also define the frequencies *f*_*rel*_ of genotypes (RR, RS, SS) of released *Wolbachia* mosquitoes (Table 2). For the simulations done by releasing *Wolbachia* susceptible mosquitoes (*w*MelBr strain), the frequency profile was *f*_*rel*_ = (0.0, 0.0, 1.0). When releasing *Wolbachia* resistant mosquitoes (*w*MelRio strain), the frequency values *f*_*rel*_ = (0.95, 0.0, 0.05) are based on the status of wild resistant mosquitoes observed in previous studies^22^.

**Table 2:** Variable parameters used in simulations.

	Insecticide intensity	Frequencies of genotypes (RR, RS, SS)
Scenario 1: Releasing susceptible *Wolbachia* mosquitoes (*w*MelBr strain) × wild resistant mosquitoes	0.0 (none), 0.4 (low), 0.7 (moderate), 0.9 (high)	(0, 0, 1)
Scenario 2: Releasing resistant *Wolbachia* mosquitoes (*w*MelRio strain) × wild resistant mosquitoes	0.0 (none), 0.4 (low), 0.7 (moderate), 0.9 (high)	(0.95, 0, 0.05)
